# Monitoring of arrhythmia and sudden death in a hemodialysis population: The CRASH-ILR Study

**DOI:** 10.1371/journal.pone.0188713

**Published:** 2017-12-14

**Authors:** Paul R. Roberts, Donah Zachariah, John M. Morgan, Arthur M. Yue, Elizabeth F. Greenwood, Patrick C. Phillips, Philip A. Kalra, Darren Green, Robert J. Lewis, Paul R. Kalra

**Affiliations:** 1 Southampton University Hospitals, Southampton, United Kingdom; 2 Portsmouth Hospitals NHS Trust, Portsmouth, United Kingdom; 3 Salford Royal Hospital, Salford, United Kingdom; Kurume University School of Medicine, JAPAN

## Abstract

**Introduction:**

It has been suggested that sudden cardiac death (SCD) contributes around 50% of cardiovascular and 27% of all-cause mortality in hemodialysis patients. The true burden of arrhythmias and arrhythmic deaths in this population, however, remains poorly characterised. Cardio Renal Arrhythmia Study in Hemodialysis (CRASH-ILR) is a prospective, implantable loop recorder single centre study of 30 established hemodialysis patients and one of the first to provide long-term ambulatory ECG monitoring.

**Methods:**

30 patients (60% male) aged 68±12 years receiving hemodialysis for 45±40 months with varied etiology (diabetes 37%, hypertension 23%) and left ventricular ejection fraction (LVEF) 55±8% received a Reveal XT implantable loop recorder (Medtronic, USA) between August 2011 and October 2014. ECG data from loop recorders were transmitted at each hemodialysis session using a remote monitoring system. Primary outcome was SCD or implantation of a (tachy or bradyarrhythmia controlling) device and secondary outcome, the development of arrhythmia necessitating medical intervention.

**Results:**

During 379,512 hours of continuous ECG monitoring (mean 12,648±9,024 hours/patient), there were 8 deaths—2 SCD and 6 due to generalised deterioration/sepsis. 5 (20%) patients had a primary outcome event (2 SCD, 3 pacemaker implantations for bradyarrhythmia). 10 (33%) patients reached an arrhythmic primary or secondary end point. Median event free survival for any arrhythmia was 2.6 years (95% confidence intervals 1.6–3.6 years).

**Conclusions:**

The findings confirm the high mortality rate seen in hemodialysis populations and contrary to initial expectations, bradyarrhythmias emerged as a common and potentially significant arrhythmic event.

## Introduction

Sudden cardiac death (SCD) is defined as an unexpected natural death from a cardiac cause within 1 hour of onset of symptoms in a person not known to have a potentially fatal condition.[1] Based on this definition, it has been suggested that SCD contributes to 50% of cardiovascular mortality and up to 27% of all-cause mortality in hemodialysis (HD) patients.[2] The purpose of the above definition of SCD is to identify arrhythmic deaths in the absence of an immediate predisposing cause. This is based on the key finding that a rapid time from onset of illness to death is a discriminator of arrhythmia as a cause of death rather than circulatory collapse.[3] Identifying deaths that are truly sudden and cardiac can be challenging, particularly in end stage kidney disease (ESKD). Deaths are often unwitnessed and, even where witnessed, there are many reasons why sudden deaths in ESKD may be due to circulatory collapse and not arrhythmia. These include rapid fluid shifts of ultra filtration, diffuse arterial calcification, loss of autonomic tone, stroke and aortic rupture.

Prior to considering potential interventions to try and reduce SCD, such as implanting cardioverter defibrillators (ICD), a number of pertinent questions should be answered. Firstly, is truly arrhythmic SCD as common in this population as reported? Studies have identified mis-representation of SCD in registries [4] as well as differences in SCD proportions following improvement of classification systems in databases such as the UK renal registry. [5] Second, what proportion of SCD in ESKD is potentially reversible as opposed to occurring during multi system failure? The hemodynamic frailty of ESKD may obfuscate any therapeutic role an ICD might have to offer in this population, particularly during intercurrent acute illness.

ICD implantation will be beneficial for some patients, but identifying this high-risk sub-group within the HD population is difficult. Whilst wide QRS-T angle, QT duration, T wave residuum, lack of heart rate variability and the presence of left ventricular hypertrophy have been identified as potential risk predictors for SCD [6–11], documenting the burden and nature of arrhythmias associated with these remains fundamental, but to do so is challenging. Ambulatory ECG recordings only give snapshots of potential arrhythmias and to date, data on the true burden of both tachy and bradyarrhythmias in this population are limited. Bradyarrhythmias may be of particular relevance in HD patients as concomitant fibrotic and calcific processes within the heart are commonplace. True SCD in a HD population needs to be characterised better to understand possible differences in phenotype from the general population with conventional SCD risk factors.

The Cardiorenal Arrhythmia Study in HD Patients using Implantable Loop Recorders (CRASH-ILR) is one of the first studies to comprehensively evaluate this high-risk population with continuous ECG monitoring without the limitations of external cardiac monitoring and spot ECGs in otherwise asymptomatic haemodialysis patients. Our study offers the longest follow up period of continuous ECG monitoring in this population to date, and we now present our findings after >379,000 hours of monitoring.

## Methods

CRASH-ILR is a prospective, loop recorder based, single centre study of 30 unselected, established HD patients. Inclusion criteria required that participants were able to give informed consent, > 18 years of age, and receiving HD for at least 90 days. The study conformed with the principles of the declaration of Helsinki and was registered with the UK clinical research network (UKCRN ID 6356) prior to the start of the study. Ethics approval was obtained from the Integrated research application system (project ID 23410). The study was also subsequently registered with ISRCTN (study ID 35846572) to meet publication requirements for PLOS ONE. The authors confirm that all on going and related trials for this drug/intervention are registered.

Patients were recruited between 24 August 2011 and 23 October 2014 from a single tertiary nephrology centre (Portsmouth) in the United Kingdom, including its satellite dialysis units. Patients were approached in their clinic by their nephrologist if considered to be eligible and capable of complying with the regular ILR downloads at each dialysis session. Demographic information, medication details and details of primary renal disease were collected. Pre and post dialysis blood results (full blood count, electrolytes) and blood pressure measurements were collected from HD sessions close to recruitment. A 12 lead electrocardiograph was performed. Detailed 2D echocardiograms were performed by a British Society of Echocardiography (BSE) accredited echocardiographer on a Phillips IE 33 machine on a non-dialysis day. Left ventricular (LV) ejection fraction was calculated by Simpson’s biplane method and LV mass estimated by LV cavity dimension and wall thickness at end-diastole. [12] LV diastolic function was assessed using mitral valve inflow and tissue doppler as per BSE guidelines.[13]

Medtronic XT loop recorders (Reveal XT 9529, Medtronic, Minneapolis, USA) were implanted in the left parasternal region. Implantable loop recorders (ILRs) are routinely used in clinical practice for the diagnosis of arrhythmias, have around a 3 year battery life, and have magnetic resonance imaging (MRI) conditional labelling for safe patient management. A Reveal XT 9529 device weighs 15g and measures 62x19x8mm. Electrocardiographic mapping was performed to identify the best position to implant the device, which was then performed under local anaesthesia with same day discharge. Device implantation was carried out on non-dialysis days.

A device typically stores 49.5 minutes of ECG data at a time (27 minutes of automatic activation triggered by pre-programmed parameters and 22.5 minutes of patient activation). Each device was remotely linked to a base unit on the dialysis unit thereby permitting transmissions up to 3 times a week where feasible. Patients were trained on how to transmit data from their device at each dialysis session via the remote monitoring CareLink® system (Medtronic, Medtronic, Minneapolis, USA). They were also educated on how to activate the device should they have a symptomatic episode (palpitations, dizziness or blackouts). Regular downloads were strongly encouraged to ensure availability of data memory on the devices at all times.

The ILRs were all programmed in the same manner with no adjustments made to sensitivity or other parameters during the course of the study. The pre-programmed criteria for automatic detection of arrhythmias were: fast ventricular tachycardia (VT) - 12/16 beats with heart rate≥200 beats per minute (bpm); VT—12 beats with heart rate≥162bpm); asystole for 3 seconds or more; bradycardia—at least 2000msec between QRS complexes (≤30bpm) for 4 beats. Symptom activation would trigger automatic recording irrespective of whether the above criteria were fulfilled and up to three 7.5 min episodes can be recorded before download is required to free up memory on the device. Reveal XTs are highly sensitive in recognising atrial fibrillation (AF), which was automatically recorded for two minutes.

Follow up was from the day of implant to death, explant, or end of battery life of the device whichever came first. Follow up data collection was rigorously performed June 2016 as per criteria above. A research link nurse at each dialysis unit was responsible for ensuring patients were able to download from their devices, and for notifying the research team of hospital admissions, transplantation or other significant medical events.

For this study, the definition of SCD included patients who were found dead having been well at the last known point of contact, as well as those dying from as an unexpected natural death from a cardiac cause, within 1 hour of onset of symptoms in the absence of a known potentially fatal condition^1^. As soon as a death was notified, direct contact was made with the relevant medical institution/personnel to ascertain circumstances of death. Devices were retrieved as soon as possible after death and analysed.

Every device download was manually scrutinised independently by two members of the research team (PCP, DZ); occurrence of ectopic activity in each event was counted, as was the occurrence of noise/under sensing of R waves. The event adjudication panel consisted of named members of the research team (PCP, PRR, PRK). Any event felt to be of clinical significance was relayed to the nephrologist involved in the patient’s care.

## Outcome and statistical analysis

The primary outcome measure was SCD or implantation of a pacing device (tachy/ bradyarrhythmia controlling device). Devices would include implantable cardioverter defibrillators (ICDs) for ventricular tachycardia or fibrillation and pacemakers for bradycardia. The secondary outcome was the development of any significant arrhythmia necessitating medical intervention (SCD, new AF, atrial flutter, non sustained VT, and other tachy/bradyarrhythmia). Patients who were known to have AF or atrial flutter at recruitment were not considered to have developed secondary outcomes if they had a further arrhythmic event identical to previously documented arrhythmias.

Descriptive statistics are presented as mean ± standard deviation for normally distributed continuous variables, median (range) for non normal distributed continuous variables, and percentage of study population for categorical variables. Between group comparisons of baseline clinical and echocardiographic parameters were made between patients who reached any primary or secondary outcome arrhythmic event versus those who did not, taking into account the relative small size of this study. Comparisons of normally distributed continuous variables were made using unpaired t-tests, and for non-parametric variables using Mann Whitney U tests. Comparisons of categorical variables (occurrence of asystole and ectopic activity on dialysis days versus non dialysis days) were made using chi square tests.

Median survival estimates were calculated using a Kaplan-Meier method. Follow up was censored at time of death, explantation of device, or most recent data upload. The sample size of this study was not powered specifically for these analyses.

## Results

30 unselected patients were recruited between 24 August 2011 and 23 October 2014; they were all receiving standard thrice-weekly 4-hour dialysis sessions. 60% were male, and the mean age was 67 ± 12 years. Mean LV ejection fraction (LVEF) as per Simpson’s biplane formula was 55 ± 8%; only one patient had LVEF <35%. Mean LV mass in females was 197 ± 30g (severe left ventricular hypertrophy as per British Society of Echocardiography guidelines) and in males 236 ± 63g (moderate left ventricular hypertrophy). A detailed outline of baseline characteristics in found in Table 1. Individual patient characteristics are provided in a supplementary file (S1 Table).

**Table 1:** Baseline characteristics of study population. Key: SBP = systolic blood pressure, DBP = diastolic blood pressure, LVEF = left ventricular ejection fraction. Continuous variable data are expressed as mean ± standard deviation except * which indicates median (range). CHA_2_DS_2_-VASc- Risk factor scoring for AF stroke risk based on the presence of Congestive heart failure, Hypertension, Age, Diabetes mellitus, Stroke, Vascular disease, Sex/female. Covariates are available at an individual level on line.

	Overall
Number	30
Follow up time (years)	1.5 ± 1.0
Clinical characteristics	
Age (years)	67.8 ± 12.1
Gender (% male)	60%
Diabetes (%)	37%
Coronary artery disease (%)	22%
CHA_2_DS_2_-VASc	2.2 ± 1.0
Beta blocker (%)	23%
Anti-coagulation (%)	7%
Dialysis parameters	
Time on dialysis (months)	45 ± 40
Pre-dialysis SBP (mmHg)	159 ± 32
Pre-dialysis DBP (mmHg)	66 ± 18
Intra-dialytic δSBP (mmHg)*	-19 (-99, +34)
Serum urea	17.3 ± 3.2
Serum creatinine	729 ± 175
Serum sodium (mmol/L)	137 ± 4
Serum potassium (mmol/L)	4.9 ± 0.5
Haemoglobin (g/dL)	118 ± 13
Platelets (x10^9^/L)	238 ± 65
ECG and echocardiography	
Resting heart rate (bpm)	73 ± 12
PR (m)	174 ± 44
QRS (ms)	102 ± 22
LVEF (%)	55 ± 8
Left atrial diameter (cm)	4.0 ± 0.4
Left ventricular mass (g)	224 ± 53
Diastolic dysfunction (%)	38%

**Fig 1:**
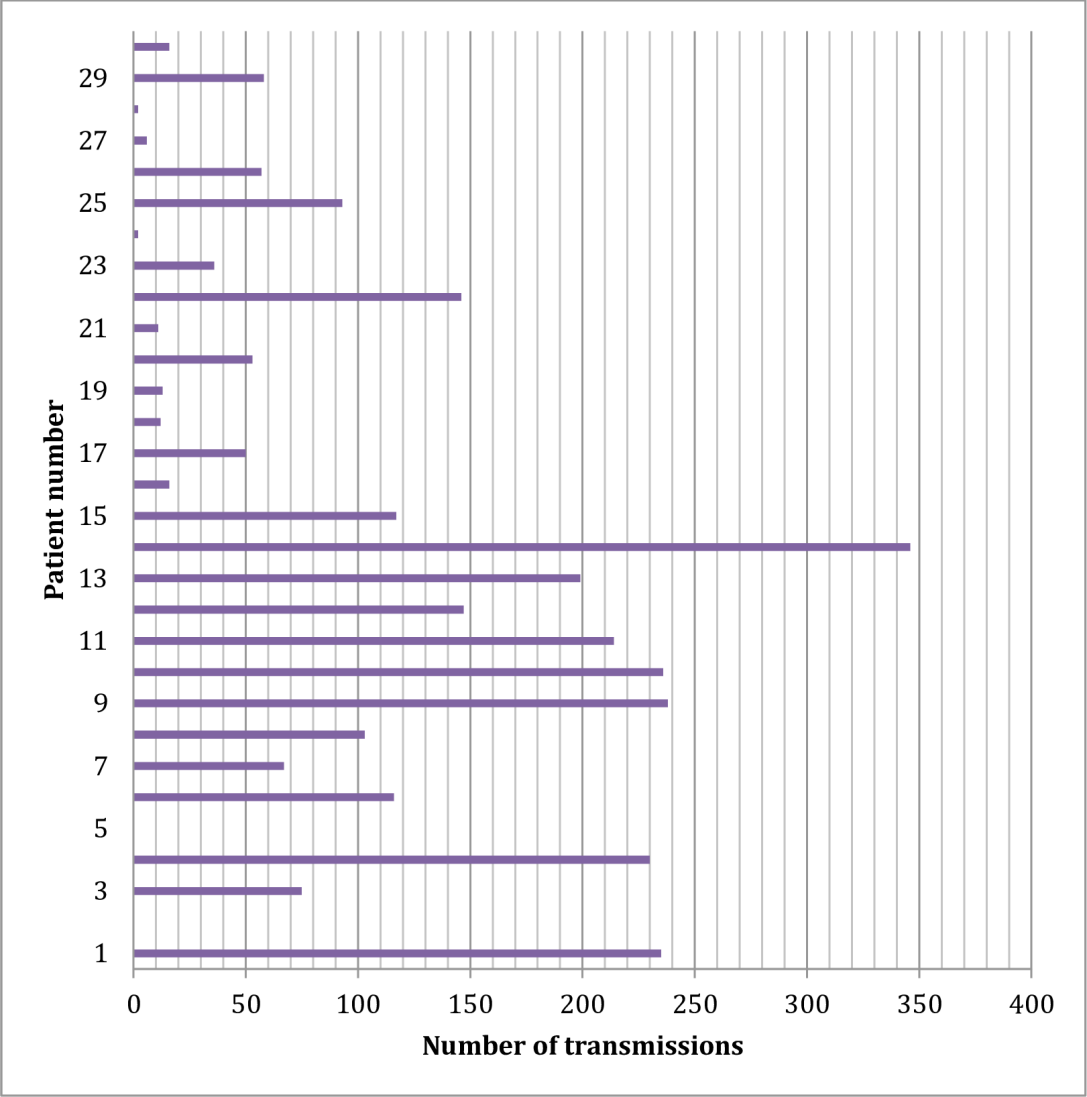
Number of transmissions per patient (patient 2 had ILR explant for infection soon after implant with no downloads prior, hence no data available. The device was misplaced following the death of patient 5 and the patient had not downloaded any data prior to death).

**Fig 2:**
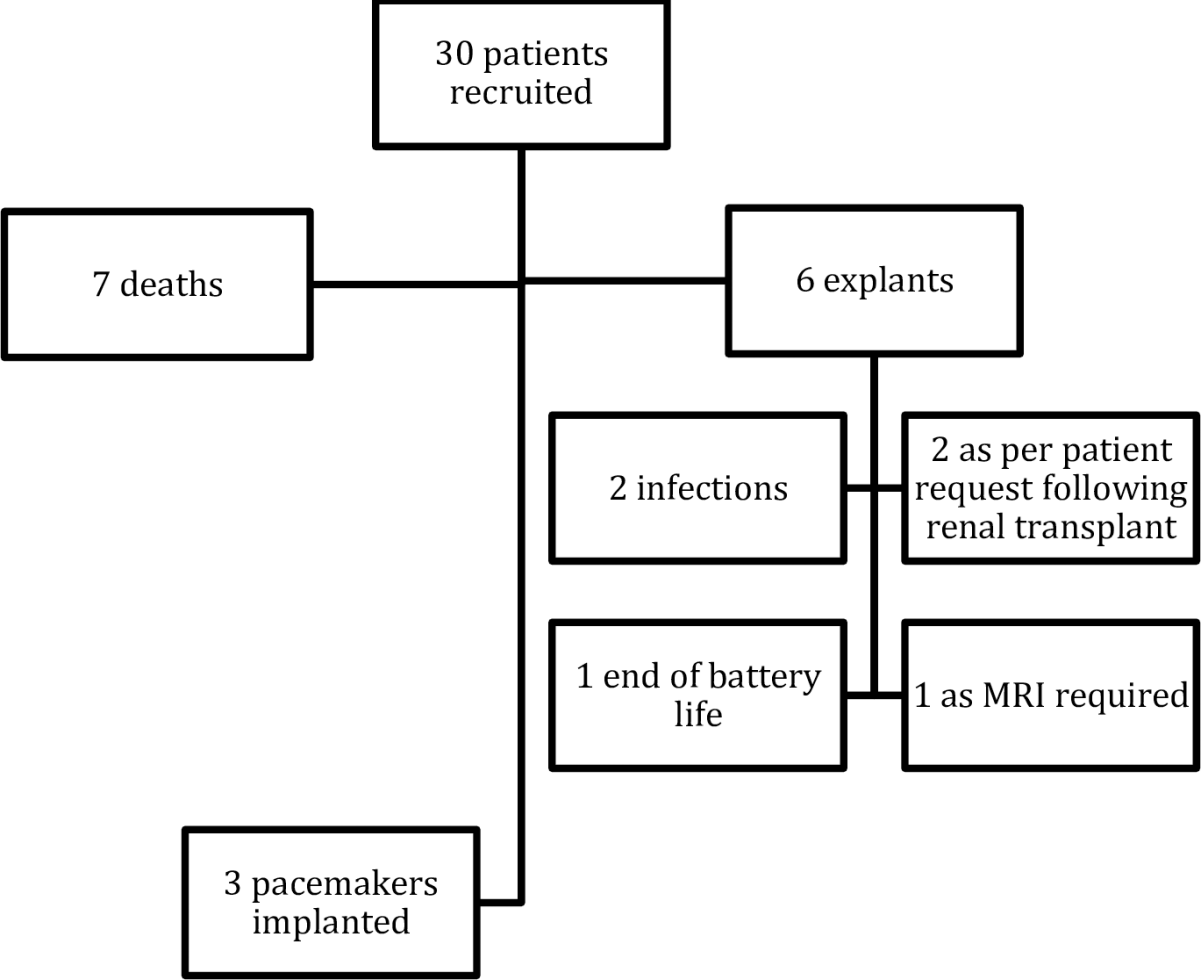
Overview of recruitment and outcomes.

The study period entailed a total of 379,512 hours (15,813 days) of continuous ECG monitoring (mean 527 ± 376 days per patient, Fig 1). During follow up, 6 devices were explanted. 2 were explanted due to persisitent superficial infection, 2 as per patient requests following renal transplant, 1 device reached end of battery life, and 1 was explanted as the patient had a clinical indication for an MRI scan and the local radiology department were unwilling to perform the scan in the presence of an ILR. 8 patients had renal transplants during the study period. Full details of recruitment and outcomes are found in Fig 2 and Table 2.

**Table 2:** Individual patient outcomes. Key: SCD- Sudden cardiac death, ILR- Implantable loop recorder, AF- Atrial fibrillation, PAF- paroxysmal atrial fibrillation, SVT- Supraventricular tachycardia, VT- Ventricular tachycardia, PPM- Pacemaker.

Study number	Age	Reason for device explant	Arrhythmic end point	Timing relative to dialysis	Outcome
1	64	-	-		ILR in situ
2	74	Infection	SCD	-	Death
3	63	Death	SCD	Mid-week	Death
4	73	-	-		ILR in situ
5	54	-	-	Weekend	Death
6	64	-	-		Death
7	83	PPM insertion	PPM	10 weekend 4 mid week	Bradycardia and PPM
8	64	End of battery life	-		Transplant
9	68	-	-		ILR in situ
10	72	-	-		ILR in situ
11	78	-	New PAF		Death
12	59	-	-		Transplant
13	56	-	-		Transplant
14	78	-	SVT	Dialysis days	ILR in situ
15	74	Transplant	-		Transplant
16	57	Death	-		Death
17	74	-	PPM		PPM
18	81	Death	-		Death
19	60	-	New PAF		ILR in situ
20	64	-	-		ILR in situ
21	36	-	-		Transplant
22	36	Transplant	VT	4 mid week 2 weekend	Transplant
23	88	-	PPM	2 mid week 1 weekend	ILR in situ
24	75	Death	Death		Death
25	75	-	-		ILR in situ
26	59	-	-		ILR in situ
27	79	MRI required	Pauses, AF	Mid week	Asymptomatic nocturnal pauses in AF
28	71	Infection	-		Infection
29	81	-	-		ILR in situ
30	74	-	-		ILR in situ

There were 17 patient activations of the ILR in 5 patients, and all of these corresponded to sinus rhythm. The symptoms leading to these activations included light headedness and feeling generally unwell.

### Primary outcome events

During the study there were eight deaths (details of deaths in Table 3). ILRs were not able to be explanted for post humous analysis in two of these patients as we were notified of their deaths only after burial. Of the other 6 deaths, two were attributable to SCD. Ventricular fibrillation (VF) was identified in one SCD patient’s post humous ILR download with evidence of coronary artery disease (CAD) on post mortem (PM). No arrhythmic events were recorded prior to the terminal arrhythmia. The other patient died from unrelated SCD, several weeks after ILR explantation for infection (no PM). (Although no downloads had been received from this patient, interrogation did not reveal any arrhythmias at the time of explant for local infection.)

**Table 3:** Deaths in CRASH–ILR; a breakdown of findings. ESKD- end stage kidney disease, IHD- Ischaemic heart disease, CAD- coronary artery disease, MI- myocardial infarction, DM- Diabetes mellitus, HTN- hypertension, COPD-Chronic obstructive pulmonary disease, CHF-chronic heart failure, PEA- pulseless electrical activity, VT- ventricular tachycardia.

Study number	Age	Device interrogated Y/N	Arrhythmia detected	Cause of death	Registered cause of death	
					Primary cause of death	Contributory causes of death
2	74	N (explanted before death)	No	Sudden cardiac death few weeks after device explanted for superficial infection. No post mortem.	ESKD	DM
3	63	Y	VF	Found dead at home.	IHD	DM. CHF
5	54	N	Device not retrieved.	Post mortem carried out.	MI	DM
6	64	Y	PEA	Admitted to hospital with GI bleed, subsequently felt to be too sick for further investigations. Post mortem carried out.	Coronary artery thrombus	HTN, COPD
9	68	Y	PEA	Death, generalised deterioration following prolonged admission	ESKD	
11	78	Y	PEA	Death	ESKD	
18	81	Y	PEA -terminal event. Self-limiting VT also seen.	Death following palliative input. Prior admission with leg ulcers requiring limb amputation.	Sepsis	ESKD
24	75	N	Device not retrieved.	Death following generalised deterioration. Died in hospice after withdrawal of dialysis.	Sepsis	ESKD

Implantable loop recording demonstrated 2:1 atrio-ventricular (AV) block and significant sinus pauses (>3 seconds) in three asymptomatic patients that resulted in pacemaker implantation (2 dual chamber devices and 1 biventricular pacemaker). The decision to implant pacemakers was made after thorough clinical assessment of the patient in the light of ambulatory ECG fndings and international guidelines. Recruitment ECG showed left bundle branch block with QRS prolongation of 172msec in the patient with AV block. The patients who received pacemakers for sinus pauses did not have any suggestions of impending heart block (PR interval prolongation, QRS widening or bundle branch block) in the run up to documentation of bradycardia. Bradycardic events were intermittent with pauses lasting between 3 to 7 seconds, and although predominantly nocturnal, all patients who had pacemakers implanted had also developed day time pauses. All patients found to be bradycardic had a comprehensive review of their medical records to ensure that there was no specific reverisble cause for the bradycardia e.g. gross electrolyte abnormality. There was no association with the long inter-dialytic period (Table 2) and there was no statistical difference in the occurrence of asystolic episodes on dialysis versus non-dialysis days (p = 0.999).

Five patients therefore reached a primary outcome event (2 SCD, 3 pacemakers). The overall event rate for primary end point was 116 per 1000 patient years. For SCD the event rate was 46 per 1000 patient years, and for device implantation 70 per 1000 patient years. Baseline characteristics that were significantly different between patients who did and did not reach a primary end point were mean age (76±8 years versus 66±12 years respectively, p = 0.04), LVEF (49 ± 10% versus 57 ± 6%, p = 0.04), and LV mass (272 ± 97g versus 213 ± 39g, p = 0.05).

### Secondary outcome events

There were 6 deaths not attributed to SCD. Two patients had confirmed CAD as cause of death following PM, (one patient had VF as the terminal event on ILR; this death occurred during hospitalisation with a severe gastro intestinal bleed; the patient was deemed too sick to have an endoscopy and death was expected. The other patient’s was not retrieved). The final four patients had generalised deterioration requiring palliative care before death with evidence of sepsis in two of these patients. One of the septic patients (study number 18, Table 3) had confirmed non sustained VT and P-wave asystole during the period of progressive decline, the second patient had gradual bradycardia and asystole and the ILR was not retrieved in the third patient.

A total of 29,435 events were identified over the study period by the reveal device in the tachycardia log, but 99.7% were due to oversensing (device interprets noise/artefact as tachyarrhythmia). Automatic detection identified 1,387 bradycardic events of which 99.5% were due to device undersensing (failure to identify electrical activity that is present). The true events consisted of sinus bradycardia, sinus pauses and AV block.

Two patients were known to have persistent AF and one had atrial flutter at the start of the study. Three patients were detected to have new onset paroxysmal AF of which two required initiation of anti-arrhythmic drugs. Two of these patients were commenced on anticoagulant therapy by their nephrologist (CHA_2_DS_2_-VASc scores 3 and 1, respectively).

A further patient (study number 22, Table 2) had recurrent slowVT documented in the context of a structurally normal heart and was initiated on beta blockers (42% of events occurring on dialysis days). Another patient who had supraventricular tachycardia (all events on dialysis days) remains under monitoring due to low burden of events. Both patients were asymptomatic. As mentioned earlier, the patient who died of a VF arrest did not have any signifcant pre-morbid events recorded via the ILR prior to death.

Ten patients reached an arrhythmic primary or secondary end point. The overall event rate for any arrhythmic end point was 232 per 1000 patient years. The estimate of median event free survival for any arrhythmia was 2.6 years (95% confidence intervals 1.6–3.6 years). Event rate for VT/VF was 46 per 1000 patient years and for new AF 83 per 1000 patient years. Event rate for bradycardia was 91 per 1000 patient years. Event rates for arrhythmias appeared to vary according to age: for example, the rate of any arrhythmic event in patients ≥65 yrs was 224 per 1000 pt years and 323 per 1000 patient years in those < 65 years (brady arrhythmia event rate: 106 per1000 pt years vs 65 per 1000 pt years; VF/VT: 0 per 1000 pt years vs 129 per 1000 pt years in patients ≥65 yrs vs patients < 65 years respectively).

**Fig 3:**
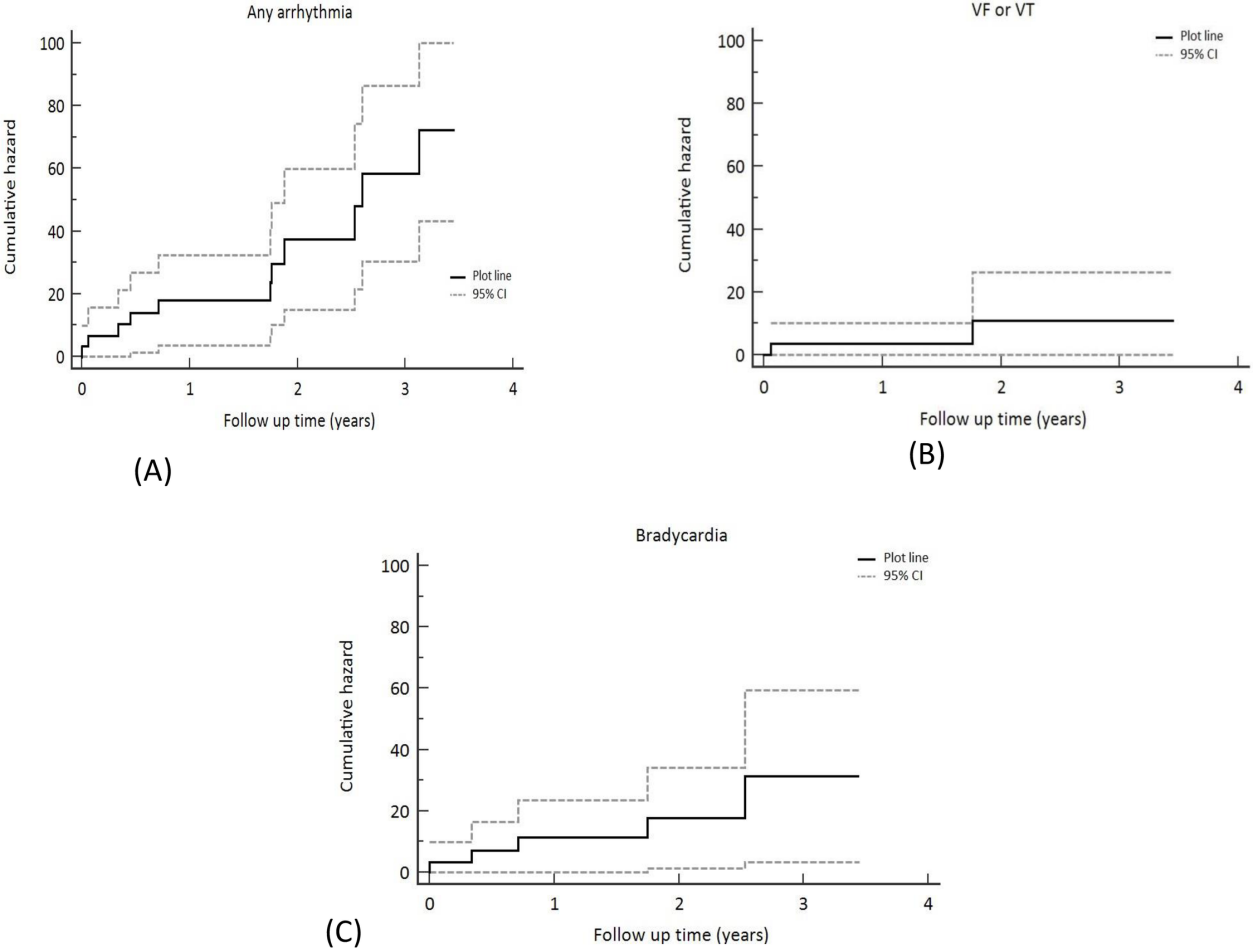
- Kaplan Meier hazard plots for (A)any arrhythmia (B)ventricular arrhythmia and (C)brady arrhythmia. (95% confidence intervals represented as dotted lines).

A Kaplan-Meier curve of time to events is shown in Fig 3. Baseline characteristics significantly different between patients who did and did not reach any arrhythmic end point were left ventricular mass (273 ± 70g versus 200 ± 28g, p< 0.01), and LVEF (52 ± 10% versus 57 ± 5%, p = 0.03). Baseline characteristics between patients with and without arrhythmia are compared in S1 Table.

### Ectopy

Ectopic activity was defined as intrinsic cardiac activity occuring from a site other than the sino atrial node at a time before the next expected intrinsic elcctrical activity. It was classified as atrial or ventricular in origin depending on p/QRS morphology and deemed significant to be counted if more than 3 ectopics occurred in a 2 minute recording.

Nine patients were identified as having signifcant ectopic activity (2 atrial, 7 ventricular). One of the patients with atrial ectopic activity had >50% of these events recorded on dialysis days. Four of the 7 patients with ventricular ectopy had >50% events on dialysis days. No statisitically siginificant difference was demonstrated between dialysis and non dialysis days for atrial or ventricular ectopy (p = 0.43 and 0.39, respectively).

### Undersensing and oversensing

Undersensing (defined as failure of the device to sense intrinsic cardiac activity) was documented in 26 of the 30 patients, predominantly due to variations in R wave amplitudes. Undersensing was significantly more on non dialysis days (p = 0.0001, 95% CI 34.2 to 78.5%)

Over sensing (defined as inappropriate recognition of signals as native cardiac activity) was documented in 24 of the 30 patients. This was predominantly due to T wave oversensing subsequent to sudden changes in T wave morphology or due to artefact/ noise.

## Discussion

Our findings confirm the high mortality rate seen in haemodialysis populations and contrary to initial expectations, bradyarrhythmias rather than tachyarrhythmias emerged as the commonest and most significant arrhythmic event. Analysis of 379,512 (median 13,356) hours of continuous ECG monitoring in our cohort suggests that, whilst arrhythmias are relatively common, a high proportion of fatal arrhythmia which may otherwise be classified as SCD occur at the end of natural life or in the context of a significant non-cardiac inter-current illness, and are therefore unlikely to benefit from preventative strategies such as ICD. Marked bradycardia, necessitating pacemaker implantation, was found in 10% of our cohort, all of whom were asymptomatic. Several observational studies have demonstrated pacing improves survival and in those who are symptomatic, prevents recurrence of syncope. Death in patients with untreated AV block can also be caused by prolonged asystole or bradycardia-triggered ventricular tachyarrhythmia. [14–17]

Supporting data towards the potential importance of bradycardia has come from a recent study by Wong et al. who evaluated 50 HD patients with ILRs for a mean of 12 ± 4 months. [18] In this study no VT/VF was documented and all 6 deaths occurred with severe bradycardia and ensuing asystole; the authors defined 83% of deaths in this study as SCD. Silva and colleagues evaluated predictors of arrhythmic events in transplant candidates with ILRs and found that out of 18 deaths, 7 were sudden cardiac events: 3 bradyarrhythmias, 1 VF, 1 myocardial infarction, and 2 undetermined. [19] CRASH-ILR, with an even more prolonged period of monitoring and with attempts made to download data at every HD session, found asymptomatic episodes of significant bradyarrhythmia in 10% and that SCD accounted for 28% of all deaths, which is similar to that noted in previous USRDS studies.

These findings are fundamental when designing studies to evaluate interventions directed towards reducing the risk of SCD. Bradycardia in HD patients remains largely undefined and could represent a manifestation of the accelerated calcific and fibrotic processes characteristic of the hearts of ESKD patients. The pacemaker implantation rate of 10% in this asymptomatic population is significantly more that the 2.5% prevalence rate seen in registry data of older persons. [20, 21] Implanting pacemakers is a cost effective and potentially lifesaving intervention and the influence of ESKD on the presence of conduction system disease needs to be tested in larger studies. It is impossible to conclude from this study whether implantation of pacemaker for asymptomatic bradycardia might contribute to reducing the risk of SCD.

Current guidelines for implantation of primary prevention ICDs require the presence of severe LV systolic dysfunction or the presence of high-risk congenital or inherited conditions. [22] Thus although up to 15% of patients have severely impaired LV systolic function at the initiation of chronic haemodialysis, [23] only around 6% of HD patients fulfil criteria for primary prevention ICDs after taking into consideration recommended factors such as life expectancy, comorbidities, functional status, presence of ischemic heart disease as well as psychological impact of having an ICD.[24] ESKD is not classified as a risk factor for SCD in its own right despite the presumed high risk. Also, the prognostic role of severely impaired LV function in ESKD has been questioned and studies have found it to be non-predictive of SCD. [25]

Where ICDs have been implanted in HD patients, there has not been clear demonstration of benefit. Pun et al identified 108 dialysis patients from the National Cardiovascular Data Registry's ICD Registry who had received primary prevention ICDs and compared them with195 dialysis patients with similar characteristics who did not have ICDs. [26] One and 3 year mortality was 42.2% and 68.8%, respectively, in the ICD registry cohort compared with 38.1% and 75.7% in the control cohort with no significant survival advantage associated with ICD [hazard ratio (HR) 0.87, 95% confidence interval (CI) 0.66–1.13, log-rank P = 0.29] with or without propensity matching.

CRASH-ILR has highlighted a number of challenges when utilising ILR technology in a HD population. Despite strict aseptic precautions at implantation, two patients developed infection requiring explant (two weeks and three months post implant respectively); this reflects the general susceptibility of dialysis patients to procedure related infections. [27] The Reveal XT is particularly sensitive for the detection of AF. Yet AF was incorrectly identified in many transmissions due to T wave over sensing resulting from intermittent deep T wave inversion, under sensing of R waves following a drop in the size of recorded QRS complexes, as well as noise/interference. The false positive auto detected episodes in CRASH-ILR were greater than previously reported rates of 22.8% for Reveal XT devices. [28] Detailed analysis of our cohort suggested greater under-sensing and over-sensing on non dialysis days, perhaps implying a role for increasing fluid volumes and reductions in thoracic impedances. As originally specified in the protocol, we did not make any adjustments to sensitivities of individual devices during the study. If ILRs were considered in clinical practice for monitoring patients on dialysis a tailored adjustment of the sensitivity would avoid a significant amount of over and under-sensing. These findings need to be carefully considered when designing larger studies and are likely to necessitate the incorporation of re-programming protocols for the ILRs after initial data download.

The patient number in this study was small making it difficult to draw generalizable conclusions about the HD population as a whole. The small sample size also limited the statistical analysis. We analysed differences in baseline characteristics between patients who did and did not reach an end point, which is less robust than a multivariate proportional hazard model in determining whether such factors are independent predictors of outcome. However, with n = 30 and 5 primary end points, type 2 error would be very likely in such a model. In a number of cases data were lost where the device could not be retrieved following death, despite extensive education of local mortuaries regarding the need to inform local pacing/device clinics when patients die with an implantable device in situ. However, the frequent downloads from the ILRs is a key strength of the study, ensuring that there was no overwriting of ECG traces and events were not missed due to lack of device memory space. Recruitment was slow due to the challenges around recruiting patients with chronic disease; close working between cardiology and nephrology colleagues was key to the success of this study. The invasive nature of cardiac monitoring was a deterrent for many patients but the introduction of much smaller new generation injectable loop recorders, (Reveal linq- Medtronic, USA) would make this less of an issue. These devices are easier to implant with low risks of infection and are better equipped to deal with sensing issues. [29, 30]

We have demonstrated that a study of ILRs with attempted downloads at every HD session is feasible in HD patients. Following almost 380,000 hours of continuous ECG monitoring in a sample cohort characteristic of a typical HD population, our findings raise pertinent issues. Undoubtedly there is a high mortality risk in patients receiving HD, yet SCD due to VT/VF is uncommon. Asymptomatic bradyarrhythmia requiring implantation of devices was common (10% of our cohort). The higher susceptibility to infection and potential risks of inappropriate shocks due to over-sensing for patients with ICD have been highlighted.

Further analysis is required to see if expanded use of ILRs in the HD population might help risk stratify those at risk of life-threatening arrhythmias and whether detection and intervention towards asymptomatic bradyarrhythmias might translate into a life prolonging intervention. Data from the US based Monitoring in Dialysis (MD) study of ILRs in HD are also awaited. [31] Many lessons have already been learnt that will inform the design and conduct of larger scale studies to identify a phenotype of HD patients that might have a modifiable risk of SCD. The value of routine use of ILRs in a dialysis population could only be justified if a prospective randomised study of normal care versus care driven by ILR information demonstrated better outcomes. An integrated approach between cardiologists and nephrologists is key.

## Supporting information

S1 Table Baseline characteristics of study population, overall and divided into those who did and did not reach an arrhythmic end point. (DOCX)

S1 File CRASH patients- baseline characteristics.xlsx. (XLSX)

S2 File CONSORT 2010 Flow Diagram. (DOC)

S3 File Protocol CRASH-ILR. (DOC)

S4 File Trend checklist. (PDF)
