# Supplementary material for: Monitoring of arrhythmia and sudden death in a hemodialysis population: The CRASH-ILR Study
Source: PLoS One. 2017 Dec 14;12(12):e0188713. doi: 10.1371/journal.pone.0188713 (PMC5730159; doi:10.1371/journal.pone.0188713)
Supplement: S1 Table — (DOCX) [file pone.0188713.s001.docx]

S1Table. Baseline characteristics of study population, overall and divided into those who did and did not reach an arrhythmic end point (sudden cardiac death, pacing device implantation, new supraventricular or ventricular brady or tachyarrhythmia), and including between group comparison p values. ^#^ indicates a significance of p<0.05. Key: SBP = systolic blood pressure, DBP = diastolic blood pressure, LVEF = left ventricular ejection fraction. Continuous variable data are expressed as mean ± standard deviation except * which indicates median (range). CHA_2_DS_2_-VASc- Risk factor scoring for AF stroke risk based on the presence of Congestive heart failure, Hypertension, Age, Diabetes mellitus, Stroke, Vascular disease, Sex/female.

|  | **Overall** | **Arrhythmia** | **No arrhythmia** | **p** |
| --- | --- | --- | --- | --- |
| Number | 30 | 8 | 22 | - |
| Follow up time (years) | 1.5 ± 1.0 | 1.3 ± 1.0 | 1.5 ± 1.0 | - |
| Clinical characteristics |  |  |  |  |
| Age (years) | 67.8 ± 12.1 | 66.5 ± 15.6 | 68.4 ± 10.7 | 0.699 |
| Gender (% male) | 60% | 67% | 57% | 0.704 |
| Diabetes (%) | 37% | 33% | 38% | 1.000 |
| Coronary artery disease (%) | 22% | 33% | 17% | 0.815 |
| CHA_2_DS_2_-VASc | 2.2 ± 1.0 | 2.3 ± 1.2 | 2.1 ± 1.0 | 0.803 |
| Beta blocker (%) | 23% | 22% | 24% | 1.000 |
| Anti-coagulation (%) | 7% | 11% | 5% | 0.514 |
| Dialysis parameters |  |  |  |  |
| Time on dialysis (months) | 45 ± 40 | 54.4 ± 46.7 | 41.4 ± 37.1 | 0.456 |
| Pre-dialysis SBP (mmHg) | 159 ± 32 | 168 ± 49 | 155 ± 22 | 0.332 |
| Pre-dialysis DBP (mmHg) | 66 ± 18 | 65 ± 27 | 67 ± 13 | 0.828 |
| Intra-dialytic δSBP (mmHg)* | -19 (-99, +34) | -31 (-99, +21) | -14 (-61,+34) | 0.157 |
| Serum urea | 17.3 ± 3.4 | 18.1 ± 3.0 | 16.9 3.5 | 0.447 |
| Serum creatinine | 729 ± 187 | 730 ± 168 | 729 ± 19 | 0.987 |
| Serum sodium (mmol/L) | 137 ± 4 | 137 ± 2 | 137 ± 5 | 0.944 |
| Serum potassium (mmol/L) | 4.9 ± 0.6 | 5.1 ± 0.7 | 4.8 ± 0.5 | 0.221 |
| Haemoglobin (g/L) | 118 ± 14 | 126 ± 14 | 116 ± 13 | 0.104 |
| Platelets (x10^9^/L) | 238 ± 74 | 205 ± 69 | 205 ± 74 | 0.195 |
| ECG and echocardiography |  |  |  |  |
| Resting heart rate (bpm) | 73 ± 14 | 75 ± 11 | 72 ± 15 | 0.632 |
| PR (m) | 174 ± 31 | 178 ± 25 | 172 ± 34 | 0.644 |
| QRS (ms) | 102 ± 23 | 112 ± 32 | 98 ± 17 | 0.160 |
| LVEF (%) | 55 ± 8 | 52 ± 11 | 57 ± 5 | 0.028^#^ |
| Left atrial diameter (cm) | 4.0 ± 0.4 | 4.0 ± 0.4 | 4.0 ± 0.4 | 0.829 |
| Left ventricular mass (g) | 224 ± 57 | 273 ± 70 | 200 ± 28 | 0.003^#^ |
| Diastolic dysfunction (%) | 38% | 25% | 44% | 0.477 |
