# Supplementary material for: Monitoring of arrhythmia and sudden death in a hemodialysis population: The CRASH-ILR Study
Source: PLoS One. 2017 Dec 14;12(12):e0188713. doi: 10.1371/journal.pone.0188713 (PMC5730159; doi:10.1371/journal.pone.0188713)
Supplement: S2 File — (DOC) [file pone.0188713.s003.doc]

**
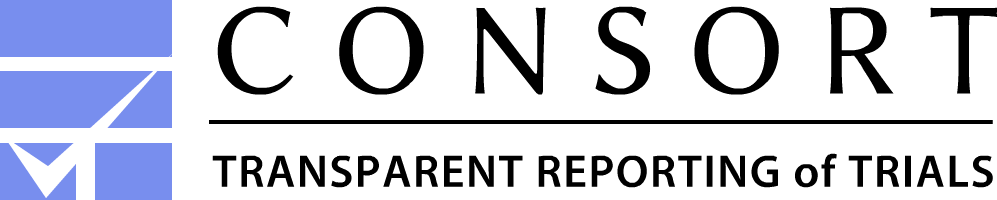
**

**CONSORT 2010 Flow Diagram**

**Allocation**

**Analysis**

**Follow-Up**

**Enrollment**

Assessed for eligibility (n= 58)

Excluded (n= 28)

♦  Not meeting inclusion criteria (n= )

♦  Declined to participate (n= 28)

♦  Other reasons (n= )

Analysed (n=30)
♦ Excluded from analysis (give reasons) (n= )

Lost to follow-up (give reasons) (n=0)

Discontinued intervention (give reasons) (n=3)

Allocated to intervention (n=30)

♦ Received allocated intervention (n=30 )

Lost to follow-up (give reasons) (n= )

Discontinued intervention (give reasons) (n= )

Allocated to intervention (n= )

♦ Received allocated intervention (n= )

♦ Did not receive allocated intervention (give reasons) (n= )

Analysed (n= )
♦ Excluded from analysis (give reasons) (n= )

Randomized (n= )
