# Supplementary material for: Monitoring of arrhythmia and sudden death in a hemodialysis population: The CRASH-ILR Study
Source: PLoS One. 2017 Dec 14;12(12):e0188713. doi: 10.1371/journal.pone.0188713 (PMC5730159; doi:10.1371/journal.pone.0188713)
Supplement: S3 File — (DOC) [file pone.0188713.s004.doc]

**Cardio Renal Arrhythmia Study in Haemodialysis patients using Implantable Loop Recorders**

**CRASH-ILR**

**(*Short or Lay Title****: Implantable Loop Recorders in Haemodialysis Patients***)**

***REC Reference No:*** 09/H0502/121

# Paul R Roberts MD FRCP

(Chief Investigator)

### Paul Kalra

### Philip Kalra

### John Morgan

(Co-Investigators)

**Chief Investigator**

Dr Paul Roberts MD FRCP

Consultant Cardiologist in Cardiac Rhythm Management, Southampton University Hospitals NHS Trust (SUHT), Wessex Cardiothoracic Unit, Mailpoint 46, Southampton General Hospital, Tremona Road, Southampton, SO16 6YD

Tel: 023 8079 4036. Emergency contact is via SUHT switchboard Tel 023 8077 7222

E-mail: [prr@hrclinic.org](mailto:prr@hrclinic.org)

**Co-Investigators**

Dr Paul Kalra, Consultant Cardiologist & Principal Investigator (PI), Portsmouth Hospitals NHS Trust (PHT)

Dr Philip Kalra, Consultant Renal Physician, Salford Royal Hospitals NHS Foundation Trust

Professor John Morgan, Consultant Cardiologist in Cardiac Rhythm Management, SUHT

#### Expert Advisors

Dr Tim Leach, Consultant Renal Physician & Clinical Director, Wessex Renal & Transplant Service, Portsmouth Hospitals NHS Trust

Dr Robert Foley, Consultant Nephrologist, Minneapolis Medical Research Foundation, Chronic Disease Research Group, Minneapolis, Minnesota, USA

#### Expert Cardiac Electrophysiologists Event Panel

Professor John Morgan, Consultant Cardiologist in Cardiac Rhythm Management, SUHT

Dr Arthur Yue, Consultant Cardiologist in Cardiac Rhythm Management, SUHT

#### Sponsor

Portsmouth Hospitals NHS Trust (PHT)

R&D Office, 1st Floor, Gloucester House, Queen Alexandra Hospital, Cosham

Portsmouth, PO6 3LY

Tel: 023 9228 6236 Fax: 023 9228 6037

E-mail [martine.cross@porthosp.nhs.uk](mailto:martine.cross@porthosp.nhs.uk)

Web: <http://www.port.ac.uk/research/nhs>

**Trial Steering Group**

Dr Paul Kalra, Consultant Cardiologist & Principal Investigator, Portsmouth Hospitals NHS Trust

Martine Cross, R&D Office, 1st Floor, Gloucester House, Queen Alexandra Hospital, Cosham, Portsmouth, PO6 3LY

**Cardio Renal Arrhythmia Study in Haemodialysis patients using Implantable Loop Recorders (CRASH-ILR)**

**(*Short or Lay Title****: Implantable Loop Recorders in Haemodialysis Patients***)**

**1. Background:**

End stage renal disease (ESRD) is the final stage of chronic kidney disease (CKD) and is defined as the failure or near failure of the kidneys to perform their normal functions. Approximately 20 million individuals in the United States (US) are affected with CKD^1^. 450,000 of these have ESRD requiring renal replacement therapy in the form of haemodialysis, continuous ambulatory peritoneal dialysis (CAPD) or renal transplantation^2^.

Patients on haemodialysis have a documented high risk of sudden cardiac death (SCD). The term SCD is commonly used to describe sudden cardiac arrest (SCA) in the setting of heart disease (although some have structurally normal hearts) with cessation of heart function, whether or not resuscitation or spontaneous reversion occurs.

Dialysis patients are at extraordinarily high risk for death. The major cause of this is cardiac disease, which accounts for around 43% of all-cause mortality. Approximately 20% of cardiac deaths in US dialysis patients are attributed to acute myocardial infarction. The single largest cause of death in dialysis patients, however is presumably as a result of serious ventricular arrhythmia, since 61% of cardiac deaths in the United States Renal Data System (USRDS) database are due to “cardiac arrest, cause unknown” or arrhythmia^3^. The cardiac arrest rate is 110/1000 patient years in the first 2 years on haemodialysis. There is a greater death rate on a Monday and an increased risk during dialysis. This risk is 7/100,000 dialysis sessions. Registry data suggests that diabetic patients on dialysis are at additional risk of SCA^4^.

A number of pathological changes occur to the heart in ESRD. There is an increased incidence of coronary artery disease, left ventricular hypertrophy and also abnormal myocardial structure. Endothelial dysfunction has been documented in addition to interstitial fibrosis and reduced coronary perfusion reserve.

Patients with significantly impaired left ventricular ejection fraction (EF) of ≤ 35% are known to be at increased risk of SCD due to ventricular arrhythmias, irrespective of the aetiology of the ventricular impairment. Uptake of implantable cardioverter defibrillator (ICD) therapy in the haemodialysis population is low in spite of a perception of a significant prevalence of impaired ventricular function in this population.

ESRD is associated with a number of pathophysiologically unusual clinical scenarios. Patients are frequently anuric which leads to significant changes in circulating volumes. This can be as much as 3-4000 ml between dialysis sessions. Additional circulating volume produces left ventricular (LV) stretch and can result in pulmonary oedema due to volume overload. During dialysis this volume may be removed over a period of 3-4 hours. This may result in hypotension and ischaemia. Patients may often be hypertensive with their renal disease or when they are fluid overloaded. A number of significant biochemical changes are observed with ESRD. There may be sudden changes in electrolyte levels. Potassium can frequently be measured at >7mmol/L before dialysis and <4mmol/L afterwards. This change occurs over the period of the dialysis i.e. a few hours. There have been changes to the measured QTc on the surface electrocardiogram (ECG) during these electrolyte changes, a prolonged QT interval having a propensity to ventricular arrhythmia.

All of these pathophysiological abnormalities have the potential to be arrhythmogenic. It is therefore likely that many of the SCDs seen in the dialysis population may be a consequence of life threatening arrhythmias.

There are limited data in the literature evaluating the arrhythmic burden of ESRD. Monitoring of patients for potentially important yet infrequent symptoms or asymptomatic non-sustained arrhythmia is clinically and practically challenging. Conventional Holter monitoring (an ambulatory ECG recording device) may provide data for 1-10 days at a time. Event monitors require patients to have symptoms, have sufficient warning to activate the monitor and to be able to activate or attach the monitor when symptomatic. Implantable loop recorders have the potential to address these limitations.

The first commercially available implantable loop recorder (Reveal®, Medtronic, Inc) was developed in the mid 1990s – designed to monitor and record the electrical activity of the heart, in order to identify an irregular heart rhythm. This device and subsequent models have required patients to be symptomatic in order to activate these devices to store the heart rhythm or ECG. Attempts at implementing automatic arrhythmia detection algorithms have had short falls. These algorithms have tended to have many false positive recordings due to either artifactual recording or undersensing. The new generation of the implantable loop recorder (Reveal XT, Medtronic, Inc) has enhanced detection algorithms, which makes its automatic detection facilities more precise. In addition it has the facility to transmit the stored information via a secure website from the patient’s home telephone line or a mobile phone link to a clinician’s computer. This means that on a regular basis any arrhythmias that have been identified by automatic detection will be transmitted and available for review at an early stage. It will also allow for clearing of the memory bins so that further recording can occur. This secure system is called the Medtronic CareLink network and it is now well established in clinical practice for device monitoring e.g. ICD and cardiac resynchronisation therapy (CRT) devices. This allows for greater data collection and improved patient experience.

This study aims to use an implantable loop recorder to record arrhythmias in a high-risk population of patients with ESRD on haemodialysis. The high event rate of SCD in dialysis patients is well documented, however identification of these arrhythmias is lacking.

**2. Methodology:**

**2.1 Study Rationale and Design**

Subjects will undergo an assessment to ensure they meet all of the inclusion and none of the exclusion criteria. After Research Ethics Committee (REC) approval of the study and appropriate local Research & Development (R&D) registration and authorisation, consent will be obtained using the approved Informed Consent form.

Subjects will have a Reveal XT device implanted under local anaesthetic and will receive by post a CareLink unit for transmission of recorded data using their home phone. Patients will be given comprehensive instruction on operation of their implanted device and communication with the CareLink network from home. Patients may prefer to download their regular data from the Reveal XT every time they dialyse from a secure mobile phone link provided by the manufacturer for use on the dialysis unit. Patients may choose which method of transmission, they would prefer for regular data downloads. Any symptomatic episodes, happening outside of the dialysis unit that concern the patient sufficiently to activate recording may need to be transmitted from home.

Collection of rhythm data is required at baseline and monthly intervals via CareLink transmissions plus additional transmissions if symptomatic. This will continue until study closure, end of device battery life or subject death/exit. Standard review of the rhythm data will still occur monthly, even though transmissions may have occurred three times a week during the previous month. Review of any known, additional and symptomatic transmissions will be prioritised to the next available working day.

Figure 1 (page 15) demonstrates the patient pathway/study flow.

**2.2 Study Objectives**

**Hypothesis:** an implantable loop recorder may identify whether certain patients who have ESRD on dialysis are at increased risk of cardiac arrhythmias.

The primary objective of this study is the documentation of ventricular arrhythmias defined as either ventricular fibrillation (VF), polymorphic ventricular tachycardia (VT) or sustained VT (>30secs).

##### Secondary Endpoints

1. Documentation of non-sustained VT (NSVT) – defined as 3 or more beats of broad complex tachycardia.
2. Documentation of atrial arrhythmias.
3. Documentation of bradyarrhythmias (AV block or pause >3.0 sec)

**2.3 Subject Selection**

It is intended that 30 haemodialysis subjects will be recruited to the study with specific criteria, as defined below. The uptake of ICD therapy in the dialysis population is perceived to be significantly less than the general population and so these subgroups merit further investigation. The clinical management of all patients (both prior to recruitment and during the study) remains at the full discretion of their usual attending nephrologist, cardiologist and primary care physician (or other specialists involved in their care).

Standard protective hygiene procedures will be performed in relation to established vascular access for dialysis, such as arterio-venous fistulas or vascular central lines. Specifically these points of access will not be utilised in this study for ILR implant.

##### Inclusion criteria

The following criteria apply to all subjects:

- Subject has ESRD
- Subject has received regular haemodialysis for a minimum of 90 days prior to study entry and is expected to continue with haemodialysis indefinitely or until renal transplantation
- Subject is at least 18 years of age
- Subject is willing and able to comply with the clinical investigational plan and willing to remain available for follow up through to study closure
- Subject is willing and able to sign and date the study Informed Consent
- Subject has necessary telephone line at their home to enable CareLink communication

Subjects of both genders must also have at least one (or more) of the following:

1. Patients with documented poor left ventricular function (LVF) as defined by an EF ≤35% on echocardiography
2. Patients with renal failure secondary to diabetes (established diagnosis of diabetes either taking regular oral hypoglycaemic agents or insulin)
3. Patients with significant left ventricular hypertrophy, (LVH) as measured via echocardiography and defined in males as >294g and females >198g. LV dimensions determined from two-dimensional guided M-mode images, using American Society of Echocardiography (ASE) recommendations/formulae^5^.

**Exclusion criteria**

The following criteria apply to all subjects:

- Subject with a myocardial infarction in the last 40 days
- Subject with an implanted ICD or pacemaker
- Subject with an implantable loop recorder
- Subject being dialysed via left-sided tunnelled line
- Subject’s life expectancy is less than one year in the opinion of the physician
- Subject is enrolled in a concurrent study
- Subject is pregnant or breastfeeding
- Subject is expected to require investigation with thoracic magnetic resonance (MR) imaging
- Patient does not have a fixed land line telephone at home

**3. Investigational Sites:**

The multicentre study will be performed at two UK sites only:

- Queen Alexandra Hospital, Portsmouth Hospitals NHS Trust (PHT), Cosham, Portsmouth, PO6 3LY
- Southampton General Hospital, Southampton University Hospitals NHS Trust (SUHT), Tremona Road, Southampton, SO16 6YD

Patients will be recruited from Portsmouth, as this is the location of the Regional Renal Unit. Southampton has the infrastructure for expert review of data from the Reveal XT devices via the CareLink network.

**4. Study Procedures:**

**4.1 Data Collection Overview**

A review of subject files is required to determine preliminary eligibility according to subject inclusion and exclusion criteria.

A study folder will be maintained for every enrolled subject.

Clinical data will be collected for baseline/enrolment (Appendix A) and 12-monthly from medical record review until the end of the study (Appendix B). CareLink data will be reviewed at monthly intervals or additionally if the patient reports symptoms that may correlate with an arrhythmia.

The Study duration will be for approximately 3 years with interim data analysis at 12 and 24 months.

Adverse events, study deviations and study exit/death will be recorded.

**4.2 Patient Informed Consent**

Prior to initiation of any study specific procedures subject signature and date of informed consent will be obtained. Informed consent will be on the site specific headed paper that will have been approved by the REC.

Potential subjects will be approached by a member of their usual healthcare team. For example, the Principal Investigator (PI), a Cardiologist at PHT will have a pool of patients under his care, who are also on dialysis from outpatient clinics. Alternatively, a Nephrologist may ask an appropriate patient whether they would like to be considered for participation in to this study and ask permission to refer their details to the PI and his/her team.

The PI will contact the patient and arrange a mutually convenient appointment, typically prior to/following a dialysis session to discuss the study further. The study schedule and procedures will be explained, any questions answered and an information sheet provided, that they can take away with them to read. Patients are given as much time as they require to decide whether they want to participate and an opportunity to ask further questions, should they arise. The potential candidate may discuss the study with their relatives, friends or GP should they wish. If the patient agrees to participate, signed informed consent is obtained.

**4.3 Baseline/Enrolment**

Baseline/enrolment data will be collected for subjects meeting all eligibility requirements. The point of enrolment is when a subject has signed the Informed Consent form. The following information is required as part of the subject’s baseline evaluation for the study and must be recorded in the patient’s study file (Appendix A):

- Inclusion and exclusion criteria
- Copy of signed consent form
- Subject demographics and medical history
- Blood tests (last recorded)
- Dialysis program details
- 12 lead ECG
- Baseline trans thoracic echocardiogram (at enrolment on day before a dialysis session) to confirm EF and LVH using standardised methodology. Patient’s eligibility confirming their poor EF and/or LVH will already have been determined from previous echocardiograms (or other imaging techniques) within their medical notes.
- Current symptoms (including NYHA classification)
- Subject physical assessment
- All medications

**4.4 Device Implantation**

The Reveal XT device will be implanted under local anaesthesia on a non-dialysis day. A standard hospital procedures consent form will be signed prior to the procedure. The devices will be implanted in a sterile environment using standard surgical implantation techniques. The Reveal will be implanted in a position on the left side of the chest wall. The devices are subcutaneous and do not have any intravascular component. They are approximately 1.5cm by 2.5cm by 0.5cm. Fully absorbable sutures will be used for skin. The exact location will be determined by the mapping techniques recommended by the manufacturer. The subject will be given the standard hospital advice information regarding wound care and suture care. Patients will receive instruction on activation of their device in the event of symptoms and will be given contact details for the process of device interrogation prior to receiving their CareLink device. For regular downloads patients may opt to download data monthly from home or every time they dialyse using a mobile phone link on the dialysis unit.

**4.5 Device Follow Up**

A wound inspection will be performed by the PI or an appropriate member of his team 5-10 days post implantation during a scheduled dialysis session. The date/time of wound inspection will be agreed with the subject at device implant.

Subjects will be issued with their CareLink interface device within 6 weeks of device implantation. Patients will receive instruction during a dialysis session on CareLink transmissions. A first test transmission will be scheduled within 4 weeks of receiving the device either from home or on the dialysis unit.

Subjects will be given a schedule of transmission dates for the future year e.g. transmission to be made within the first seven days of the calendar month, if they opt to use their home telephone line.

Whichever route of transmission is chosen – patients will be contacted initially by telephone if the transmission has failed or they have failed to make a transmission. If the patient cannot be contacted by phone, then a letter will be posted asking them to perform another transmission. Patients will receive by post or telephone notification of a successful transmission and the outcome of that transmission. This will be:

- No clinically significant arrhythmias

Or

- Clinically significant arrhythmias – further action required

If subjects have symptoms and have activated their device they can then make a transmission and inform the research co-ordinator or PI. The data will be viewed and feedback by telephone or post made to the subject.

All data transmitted to CareLink will be reviewed initially, by either of the cardiologists, Dr Paul Roberts (CI) or Dr Paul Kalra (PI). Once the study is established, it may be possible to delegate review of downloads to certain staff, for example Cardiology research registrars. Any events determined to be clinically significant will be reviewed by either Dr Paul Roberts or Dr Paul Kalra and the patient’s responsible clinician informed. Any events identified to be clinically significant will be treated according to normal clinical practice. Appendix C will be completed for documentation of arrhythmic and/or other clinical events.

All events determined to be arrhythmias will be independently reviewed by a panel of expert Cardiac Electrophysiologists (Professor John Morgan & Dr Arthur Yue). They will not have any clinical data on the subject and will be unaware of whether the arrhythmia was associated with symptoms or recorded as a consequence of automatic detection. This panel will meet 6 monthly to analyse clinical events and a report completed for each event.

**4.6 Subject Follow Up**

There will be an annual review of the subjects’ clinical status, recorded on Appendix B and filed in the subject’s research folder. Any major clinical event in the interim will also be recorded e.g. hospitalisation, cardiac event, death on Appendix C.

The dialysis records for all patients will also be obtained if a clinically significant cardiac event is established, to ensure for example adequate dialysis, vital statistics such as blood pressure, bloods.

**4.7 Study Duration**

The 30 patients for Reveal XT implant will be recruited over a period of approximately 6 months.

The study will continue until the implanted device has reached the end of its battery life. The projected longevity of these devices is approximately 3 years. When this point has been reached the subject will be scheduled for device explantation. This will be performed after a dialysis session in sterile conditions using local anaesthesia. The study will close when the last patients’ implanted device has been explanted.

**4.8 Study Exit**

A study exit will be documented for any of the following:

- Subject death
- Study completion (approximately 3 years after device implant)
- Subject withdrawal/lost to follow up
- Investigator withdrawal of the subject

*Subject Death:*

In the event of a subject death, the following information related to the death will be documented:

- Date of death
- Date research co-ordinator became aware of death
- Primary cause of death
- Death classification
- If death was a result of an arrhythmic death

In all cases of a subject death every attempt should be made to interrogate the implanted device. In all cases the mortuary or funeral home needs informing of the implanted device. In the case of the subject being cremated the device must be explanted first. In the case of a burial ideally the device should be explanted. If this is not possible then all attempts to interrogate the device prior to burial should be made. Once explanted, all events recorded on the device should be interrogated prior to device disposal.

*Subject Withdrawal/Lost to Follow Up:*

A subject may withdraw from the study by notifying the study investigator. This should be recorded in the patient’s study folder (Appendix C) and the reason for withdrawal indicated.

If a subject recurrently defaults follow up transmissions in spite of contact with the study investigators or co-ordinators then they should be considered for study withdrawal.

*Investigator Withdrawal of Subject:*

If the investigator chooses to withdraw a subject from the study, the investigator must provide documentation to the subject stating the reason why the subject was withdrawn. In the event of an infected implanted device the device will need to be explanted in the usual manner and the patient withdrawn from the study.

On withdrawal of a patient from the study a suitable time will be arranged to have the device explanted in a standard sterile fashion.

**4.9 Safety Reporting and Serious Adverse Event (SAE)**

Adverse events will be recorded in accordance with PHT Research Related Adverse Event Reporting Policy. An SAE is classified as an adverse event that is a direct consequence of the subject’s participation in the study leading to:

1. Death
2. Serious deterioration in the health of the subject that:
   - Resulted in a life-threatening illness or injury
   - Resulted in permanent impairment of body structure or function
   - Required “in patient” hospitalisation or prolongation of existing hospitalisation
   - Resulted in medical or surgical intervention to prevent permanent impairment to body structure or a body function

A Trial Steering Group consisting as a minimum of Dr Paul Kalra and Martine Cross, as Sponsor representative will meet 3-6 monthly as necessary throughout the study to review recruitment and any SAEs. Other Investigators/ expert advisors / co-ordinators may also attend these meetings, as required. Appendix C will be completed for review of any clinical events at Trial Steering Group meetings.

**5. Statistical Methods and Data Analysis:**

This study is observational in design. Descriptive statistical analysis (mean, median, standard deviation) will be performed on the baseline demographic data. Documented arrhythmias will be reported on an individual/collective basis.

**6. Finance and Resources:**

This research is supported by Medtronic Ltd, who have agreed to provide

1. 30 Reveal XT devices, CareLink enabled free of charge
2. The sum of £10,000

This has been allocated to reimburse patient travel costs for study attendance, on for example non-dialysis days and to support the following research activities:

12-lead ECG, echo, device implant & programming and explant: Performed by PI (as part of SPA time). Review of CareLink downloads by PI, CI and Events Panel (as part of SPA time). Some of these activities may be delegated to appropriate Cardiology research registrar.

Research Assistance/Administration:

No nursing input is required.

Assistance from Cardiology research registrar or Renal registrar (as part of protected MSc time) may be requested to assist the PI

A research co-ordinator at SUHT to co-ordinate meetings, receive messages from subjects who have performed extra downloads and prepare annual progress reports/end of study declarations etc

No radiology required, no cardiac technician input required, no impact on NHS lab time and the cost of devices is zero.

Consumables:

Costs of sterile kit, drapes used at implant and explant are £24.60 per case (ordered via PHT cardiology dept). Hence, an approximate total of £1,500 expenditure on consumables will be required for 30 subjects recruited to be implanted & explanted.

Patient travel costs (estimated at maximum £500 total for study - implant and explant)

**7. Monitoring and Audit:**

The study will be monitored and audited in accordance with PHT procedures. All trial and related documents will be made available on request for monitoring and audit by PHT, the relevant REC or other licensing bodies.

**8. Data Protection:**

Data will be collected and retained in accordance with Data Protection Act 1998.

Subjects will be assigned a sequential study number at the time of enrolment into the study. Each subject name, date of birth and their assigned study number will be kept as a single list on Dr Paul Kalra’s password protected NHS computer. This list will be printed and filed for archiving at the end of the study, for the required length of time, with the anonymised study files. The signed consent forms will also be stored but no other personally identifiable data will be archived.

The electronic cardiac data transmissions will be reviewed remotely by investigators and/or the cardiac electrophysiologists’ event panel using their usual CareLink password protected access, as per usual NHS practice. Dr Paul Kalra will provide the list of patient names requiring data download at the event panel or annual review and at the end of the meeting, this list will be returned or destroyed.

# 9. Storage of Records:

Study documents (paper and electronic) will be retained in a secure location during & after the trial has finished. All essential documents and source data, including any medical records where entries related to the research have been made; will be retained for a period of 10 years following the end of the study. Although the UK's Statutory Instrument for Medical Devices states 5 years for the storage of research work ([http://www.opsi.gov.uk/si/si2002/20020618.htm#16](http://www.opsi.gov.uk/si/si2002/20020618.htm#16)), the sponsor for this study has advised10 years. A ‘DO NOT DESTROY’ label stating the time after which the documents can be destroyed will be placed on the outer cover of relevant medical records.

# 10. Indemnity:

This is an NHS-sponsored research study. For NHS sponsored research HSG(96)48 reference no. 2 applies. If there is negligent harm during the clinical trial when the NHS owes a duty of care to the person harmed, NHS indemnity covers NHS staff, medical academic staff with honorary contracts and those conducting the trial. NHS indemnity does not offer no-fault compensation and is unable to agree in advance to pay compensation for non-negligent harm. Ex-gratia payments may be considered in the case of a claim.

# 11. Ethics & R&D Approvals:

The study will be performed subject to any provisions of favourable opinion from the Research Ethics Committee (REC) and Site-Specific Assessment by local Research & Development (R&D).

# 12. Research Governance Statement:

This study will be conducted in accordance with Research Governance Framework for Health & Social Care (2005) and Good Clinical Practice.

**13. References:**

1. Chronic Kidney Disease Workgroup. Kidney disease outcomes quality initiative clinical practice guidelines. *Am J Kidney Dis 2002; 39: S17-S31.*
2. US Renal Data System. *USRDS 2005 Annual Data Report: Atlas of End-Stage Renal Disease in the United States.* Bethesda, MD: National Institutes of Health, National Institute of Diabetes and Digestive and Kidney Diseases; 2005.
3. US Renal Data System. *USRDS 2006 Annual Data Report: Atlas of End-Stage Renal Disease in the United States.* Bethesda, MD: National Institutes of Health, National Institute of Diabetes and Digestive and Kidney Diseases; 2006.
4. Herzog CA. Cardiac Arrest in dialysis patients: Approaches to alter an abysmal outcome. *Kidney Int Suppl 2003: S197-S200*
5. Lang RM, Bierig M, Devereux RB *et al.* Recommendations for chamber quantification: a report from the American Society of Echocardiography's Guidelines and Standards Committee and the Chamber Quantification Writing Group, developed in conjunction with the European Association of Echocardiography, a branch of the European Society of Cardiology. *J. Am. Soc. Echocardiogr 2005: 18(12), 1440-1463*

**Figure 1: CRASH-ILR Patient Pathway/Study Flow**

Potential haemodialysis subject identified:

(EF<35% &/or ESRF 2° to diabetes &/or LVH known from medical history)

**Yes**

Exclusion criteria present?

**No**

Subject consents /signs informed consent

**Yes – follow direction of arrows**

Complete ECG (3mins) on subject

Complete baseline trans thoracic echocardiogram on a day prior to dialysis to confirm cardiac structure /status on subject (10 mins)

NB: If not diabetic, EF>35% &/ no significant LVH – then subject withdrawn and no follow-up required

Complete ILR, Reveal XT implant on a non –dialysis day for subject and device programming (15-20 mins)

Complete review of wound site 5-10 days post implant on a dialysis day for subject (5 mins)

Subject downloads cardiac ILR data every month (and if symptomatic) using CareLink Network via home phone or the regular data can be downloaded every time they dialyse using mobile phone link on dialysis unit . This continues for approx 3 years until study closure (5-6 mins/download)

Investigators review subject transmission every month and when notified of extra downloads using CareLink Network website for approx 3 years until study closure (4-5 mins/download)

Trial Steering Group convene every 3-6 months (as needed) to review recruitment and SAEs. Expert cardiac electrophysiologist panel review of any events every 6-months (if needed) to independently confirm arrhythmia for approx 3 years or until study closure

Annual review of the subjects’ clinical status by Investigator

Subject exit from study occurs when:

- ILR reaches end of battery life. Explant scheduled following dialysis session(10 mins)
- Subject death or withdrawal/lost to follow-up
- Investigator withdrawal of the subject

Study closure when the last patients’ implanted ILR device has been explanted
